# U-shaped association between BMI and cognitive impairment in middle-aged and older adults with type 2 diabetes: effect modification by lifestyle and exercise

**DOI:** 10.3389/fpubh.2025.1675383

**Published:** 2025-12-01

**Authors:** Juan Ge, Yuqin Han, Shuzhi Peng, Shan Zhang, Limei Zheng

**Affiliations:** 1 College of Health Management, Shanghai Jian Qiao University, Shanghai, China; 2 Shanghai Fengxian District Central Hospital, Shanghai, China

**Keywords:** diabetes, cognitive impairment, body mass index (BMI), nonlinear association, restricted cubic splines (RCS), cross-sectional study

## Abstract

**Objective:**

To explore the correlation between body mass index (BMI) and cognitive impairment in type 2 diabetes patients through a cross-sectional observational study.

**Methods:**

Data on basic information and cognitive impairment of type 2 diabetes were collected through questionnaires, and the correlation between BMI and cognitive impairment of type 2 diabetes was analyzed using logistic regression model, restricted cubic spline (RCS) model and subgroup analysis. At the same time, the interaction between BMI and exercise, living status and other factors was tested.

**Results:**

A total of 565 valid samples were included in this study and 51.15% had cognitive impairment. The mean BMI score was 24.51 ± 2.16 kg/m^2^. An inverse association between BMI score and cognitive impairment in patients with was observed in all three models. Subsequent regression analysis using RCS confirmed this nonlinear association and found two inflection points at 23.72 kg/m^2^ and 27.77 kg/m^2^. Specifically, cognitive impairment increased with decreasing BMI at BMI scores <23.72 kg/m^2^, was least expressed in the interval 23.72–27.77 kg/m^2^, and increased with increasing BMI scores >27.77 kg/m^2^. In addition, the interaction between BMI and factors such as exercise and lifestyle was examined, and the results showed that the interaction did not reach the statistical significance level.

**Conclusion:**

Observations indicate that the U-shaped relationship between cognitive impairment and BMI observed in middle-aged and older adults with type 2 diabetes was more pronounced in those who live alone and are physically inactive. Although the interaction test was not significant, the subgroup analysis suggested that middle-aged and older adults with type 2 diabetes who live alone and are physically inactive may need to manage their BMI more rigorously.

## 1 Introduction

According to the International Diabetes Federation, approximately 600 million people worldwide currently have diabetes, and over 90% of them have type 2 diabetes mellitus (T2DM) (1). The spread of diabetes in China has reached 11.9%, with a significant correlation to age (2). T2DM is most prevalent among middle-aged and older adults, with a prevalence rate of over 30% in those aged 40 and above, which is significantly higher than in other age groups (3). This data highlights the importance of the middle-aged and older adults in preventing and controlling T2DM, and indicates that researching the health issues affecting this group is highly practical.

T2DM is not an isolated metabolic disorder. Its effects on multiple systems are well documented, with cognitive impairment being one of the most serious yet neglected complications (4). China bears the world’s highest burden of diabetes, with cases surging to 118 million today (2), where cognitive impairment affects 34.7% of older adults with T2DM (5). Numerous epidemiological studies have shown that cognitive impairment is highly prevalent among patients with T2DM (6–9). A meta-analysis of studies found that individuals diagnosed with T2DM have been shown to have a 1.25–1.91 times increased risk of developing cognitive impairment when compared to those who do not have diabetes (10). This ‘metabolic-cognitive’ comorbidity is not coincidental. Pathological processes such as microangiopathy, insulin resistance, increased oxidative stress and inflammation are induced by prolonged hyperglycaemia. These processes continue to damage the neurons and vascular system of the brain, progressively undermining the structural and functional basis of cognitive function (11). Cognitive impairment inversely impacts the quality of life and health outcomes of middle-aged and older adults with T2DM. Mild cognitive impairment can lead to memory loss, decreased executive function and difficulty with daily self-management (e.g., taking regular medication and monitoring blood glucose levels), which can exacerbate diabetes management (12). After progression to dementia, patients lose the ability to live independently. This not only causes great physical and psychological distress, but also places a significant care burden on families and society (13).

For middle-aged and older adults with T2DM, identifying the factors that influence cognitive impairment and developing targeted prevention strategies are core aspects of comprehensive diabetes management. While the association between body mass index (BMI), a metabolic indicator reflecting the relationship between body weight and height, and metabolic health is well documented (14), its role in cognitive function remains controversial (15–17). While some studies have suggested a linear association, with low or high BMI being associated with decreased cognitive functioning (18, 19), others have proposed a nonlinear hypothesis, suggesting that specific BMI intervals are associated with cognitive advantages (20).

The moderating role of lifestyle and exercise habits: recent studies have demonstrated that these factors may significantly influence the relationship between BMI and cognitive function (21, 22). Regular physical activity has been shown to enhance cognitive performance and reduce the risk of cognitive impairment (22). Additionally, non-solo lifestyle are associated with better cognitive health (23). However, most of these studies have focused on the genera middle-aged and older adults, and how lifestyle and exercise habits affect the relationship between BMI and cognitive function in middle-aged and older adults with T2DM has not been fully explored.

In addition, previous studies have not sufficiently explored the nonlinear relationship between BMI and cognitive function in patients with T2DM. This is particularly true about interaction analyses of different age groups, lifestyles (e.g., exercise habits) and social environments (e.g., residential status). This makes it difficult to reveal the heterogeneity of the association pattern. This hinders the provision of a precise basis for the cognitive health management of middle-aged and older adults with T2DM in clinical practice. Given the importance of lifestyle and exercise habits in patients with T2DM, this study specifically focuses on how these factors modulate the relationship between BMI and cognitive impairment. This study investigated BMI and cognitive dysfunction in middle-aged and older adults with T2DM, using Restricted Cubic Spline (RCS) modeling to systematically correlate the two in a nonlinear manner and subgroup analyses to reveal the potential influencing factors, aiming to provide evidence for precision management of cognitive health in this patient group.

In order to ensure the scientific nature of the research and the reliability of the theoretical basis, we prioritized peer-reviewed journals indexed by the Web of Science during the literature search, and ensured the rigor of knowledge through high-impact literature sources.

## 2 Materials and methods

### 2.1 Participants

The present study was conducted between March and June 2025 on patients with T2DM attending community hospitals in Shanghai Municipality. The selection criteria were as follows: (1) The age of the subject is greater than or equal to 40 years. (2) Voluntarily participating in the investigation and completing an informed consent form. The exclusion criteria encompassed the following stipulations: (1) Diabetic ketoacidosis or hyperosmolar hyperglycemic syndrome; (2) Severe mental disorder; (3) Severevisual or hearing impairment; (4) Central nervous system disease (e.g., stroke, Alzheimer’s disease, Parkinson’s disease); (5) withdrawal during the study; (6) Key information (e.g., age, sex, height, weight) were incomplete.

### 2.2 Instruments and measurements

The researchers employed a validated, self-administered demographic questionnaire to systematically collect data on baseline characteristics, encompassing socio-demographic variables such as educational attainment, biomedical characteristics (age, gender, height, weight and other relevant indicators). The Pittsburgh Sleep Quality Index (PSQI) scale (24) and the Centre for Epidemiological Studies Depression Scale (CES-D) (25) were utilized to assess sleep quality and depression, respectively. Cognitive function was evaluated using the Montreal Cognitive Assessment Scale (MoCA) (26), with a score of ≥26 being classified as normal cognitive function and <26 as having cognitive impairment. All scales are in Chinese and have been validated. The definitive diagnosis of T2DM was established based on the metabolic disease classification criteria published by the World Health Organization (WHO) in 1999 (27). The BMI is calculated by dividing a person’s weight in kilograms by the square of their height in meters. Weight and height were measured by trained clinical staff using a height and weight scale (instrument model: HNH-318). Participants wore light clothing and no shoes. The measurements were repeated twice and averaged. Assessments were conducted by trained registered nurses in standardized settings. Instruments were administered face-to-face in quiet rooms, with provisions for verbal clarification to ensure comprehension, minimizing measurement variability. To reduce recall bias and enhance comprehension, interviews included simplified explanations for literacy-limited individuals.

### 2.3 Sampling method

**Figure 1:**
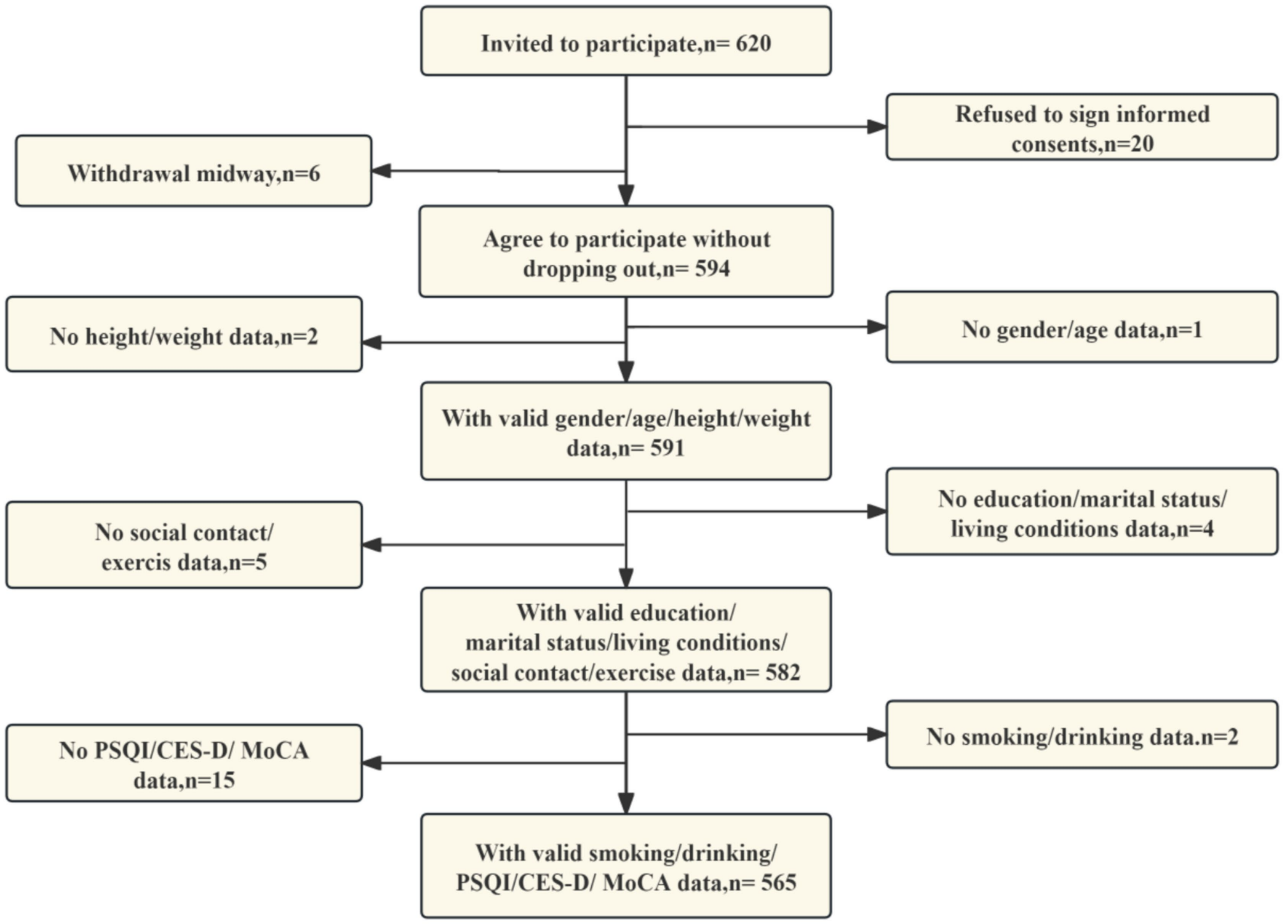
Study flowchart.

Participants were consecutively enrolled using convenience-based sampling from community hospitals. The sample size was calculated using the standard proportionality estimation formula *n* = *Z*^2^ × p(1 − p)/e^2^ with the following parameter settings: *p* = 0.15 (based on a 15% prevalence of cognitive impairment among patients with type 2 diabetes in a previous study (28)), *Z* = 1.96 (at a 95% confidence level), and a margin of error value of 5% to account for uncertainty. In order to enhance the robustness of the study, the initial sample size was increased by 20%, a decision which was based on the analysis of attrition rates and missing data. It was determined that, in consideration of the parameters above, the final minimum required sample size would be 236 respondents. The process of subject collection is illustrated in Figure 1.

### 2.4 Statistical analysis

In this study, subjects were grouped according to the presence or absence of cognitive impairment. Normality of all continuous variables was assessed by the Shapiro–Wilk test. Continuous data are expressed as the mean ± standard error (SE), whereas categorical data are shown in the form of percentages. The analysis of between-group differences in baseline variables was conducted using either the t-test or the chi-square test, depending on the nature of the variable in question. To evaluate the factors influencing cognitive impairment, **t**he findings of the present study were presented in the form of the ratio of ratios (OR) and their 95% confidence intervals (CI) using a binary logistic regression model. Three distinct models were constructed for analysis. The first of these was an unadjusted model. The second was a model adjusted for age, education, living conditions, and marital status. The third was a further adjusted model, which was adjusted for exercise, social contact, smoking, CES-D, and PSQI. These adjustments were made based on the second model. Subgroup analyses were performed to systematically evaluate the variation in the association between BMI and cognitive impairment among patients with T2DM across different populations. These variables are thought to influence the occurrence and progression of cognitive impairment (29–32). To reveal potential effect modification effects, subgroups will be divided based on sex, age (subgroups of those under 60 years and those aged 60 years and over), lifestyle (i.e., whether they live alone, yes/no), weekly exercise (i.e., whether they exercise weekly, yes/no) and literacy (i.e., whether they are illiterate, yes/no). These subgroup variables were selected based on factors associated with cognitive impairment identified in previous studies and the distribution of data from this study (31, 32). The assessment of nonlinear relationships was conducted through the implementation of the RCS test (33, 34). Statistical analyses were performed using R software (version 4.3.3), with the two-tailed *p*-value threshold for statistical significance set at 0.05.

## 3 Results

### 3.1 General characteristics of the object of study

The present study analyzed a total of 565 patients diagnosed with T2DM, with a mean age of 64.77 ± 11.50 years, and 40.71% of whom were female. The demographic characteristics of the participants are outlined in Table 1. Of the overall population, 289 (51.15%) had been diagnosed with cognitive impairment. A comparison of the cognitively impaired population with the non-cognitively impaired population revealed that the former was generally older, less educated, and more often widowed or divorced. Furthermore, the group exhibited suboptimal sleep quality, elevated risk of depression, low BMI, solitary living arrangements, infrequent participation in physical activity, diminished social engagement, and a higher proportion of smokers. Please refer to Table 1 for further information.

**Table 1:** General characteristics of the object of study.

Characteristics	Overall	Cognitive impairment		*p*-value
		Without	With	
*n*	565	276 (48.85)	289 (51.15)	
Gender				
Female	230(40.71)	104 (37.96)	126 (43.30)	0.152
Male	335 (59.29)	172 (62.77)	163 (56.01)	
BMI	24.51 ± 2.16	24.82 ± 1.74	24.21 ± 2.50	**<0.01**
Age, years	64.77 ± 11.50	62.28 ± 10.75	67.15 ± 11.71	**<0.01**
PSQI	11.78 ± 2.10	11.22 ± 2.12	12.31 ± 1.90	**<0.01**
CES-D	11.13 ± 1.94	10.76 ± 1.87	11.47 ± 1.94	**<0.01**
Marital status, *n* (%)				**<0.01**
Married	413 (73.10)	223 (81.39)	190 (65.29)	
Never married	7 (1.24)	1 (0.04)	6 (2.06)	
Divorced	31 (5.49)	11 (4.01)	20 (6.87)	
Widowed	114 (20.18)	41 (14.96)	73 (25.09)	
Education, *n* (%)				**<0.01**
Illiterate	128 (22.65)	32 (11.68)	96 (32.99)	
Primary school	142 (25.13)	54 (19.71)	88 (30.24)	
Middle school	180 (31.86)	111 (40.51)	69 (23.71)	
High school	85 (15.04)	59 (21.53)	26 (8.93)	
College	25 (4.42)	16 (5.84)	9 (3.09)	
Postgraduate	5 (0.88)	4 (1.46)	1 (0.34)	
Living conditions				**<0.01**
Living alone	80 (14.16)	28 (10.22)	52 (17.87)	
Live with family	469 (83.01)	248 (90.51)	221 (75.95)	
Living with caregivers	16 (2.83)	0 (0.00)	16 (5.50)	
Social contact				**<0.01**
Every day	59 (10.44)	31 (11.31)	28 (9.62)	
5–6 times a week	125 (22.12)	60 (21.90)	65 (22.34)	
3–4 times a week	243 (43.01)	117 (42.70)	126 (43.30)	
1–2 times a week	115 (20.35)	65 (23.72)	50 (17.18)	
Never	23 (4.07)	3 (1.09)	20 (6.87)	
Exercise				**<0.01**
Everyday	80 (14.16)	42 (15.33)	38 (13.06)	
At least once a week	123 (21.77)	62 (22.63)	61 (20.96)	
At least once a month	244 (43.19)	123 (44.89)	121 (41.58)	
Sometimes	82 (14.51)	48 (17.52)	34 (11.68)	
Never	36 (6.37)	1 (0.36)	35 (12.03)	
Smoking				**0.011**
Never smoking	435 (77.00)	225 (82.12)	210 (72.16)	
Current smoking	127 (22.48)	51 (18.61)	76 (26.12)	
Former smoking	3 (0.53)	0 (0.00)	3 (1.03)	
Drinking				0.601
Never drinking	450 (79.65)	220 (80.29)	230 (79.04)	
Current drinking	113 (20.00)	56 (20.44)	57 (19.59)	
Former drinking	2 (0.35)	0 (0.00)	2 (0.69)	

Data are expressed as mean (SD) or *n* (%).The bold values indicates statistically significant differences.

### 3.2 Association between BMI and cognitive impairment

**Figure 2:**
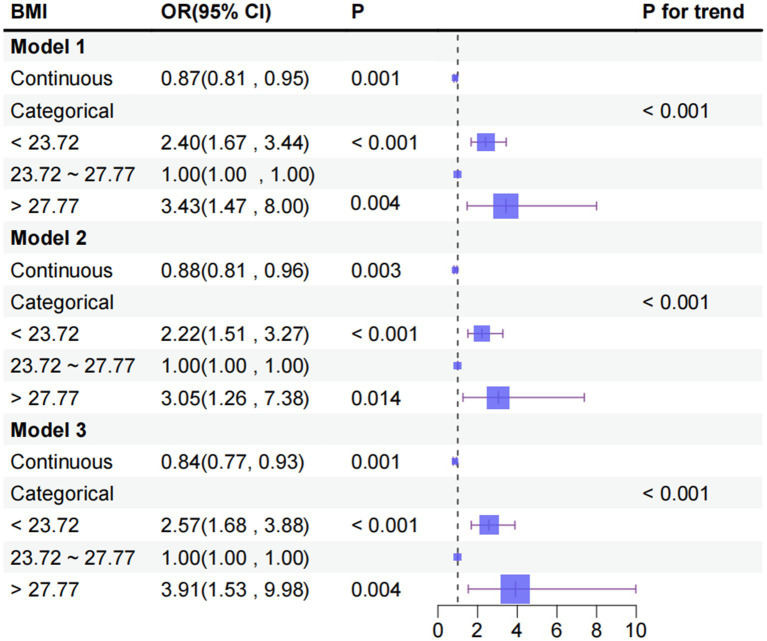
Association between BMI and cognitive impairment in T2DM. The original continuous variable of BMI was then transformed into three separate categories, based on inflection points identified through RCS. Model 1: crude model; Model 2: adjusting age; education; living conditions; marital status; Model 3: model 2 + additional adjusting exercise; social contact; smoking; CES-D; PSQI.

Multi-model regression analyses showed a significant negative association with cognitive impairment when BMI was used as a continuous variable. Statistical significance was maintained in model 1, unadjusted for covariates (OR = 0.87, 95% CI: 0.81–0.95), in partially adjusted model 2 (OR = 0.88, 95% CI: 0.81–0.96) and in model 3, fully adjusted for confounders (OR = 0.84, 95% CI: 0.77–0.93). As calculated by model 3, the occurrence of cognitive impairment was reduced by 16% for each 1-unit increase in BMI (*p* < 0.01) (Figure 2).

### 3.3 Association between BMI and cognitive impairment in a dose-dependent manner

**Figure 3:**
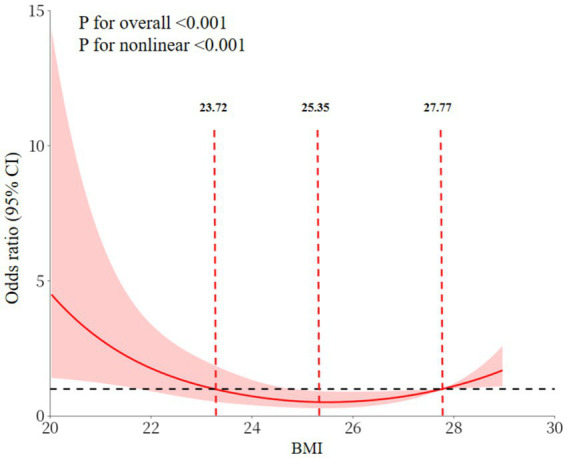
Association between BMI and cognitive impairment in a dose-dependent manner.

RCS analyses revealed a significant U-shaped association between BMI and the prevalence of cognitive impairment (overall *p* < 0.001, nonlinear *p* < 0.001). Specifically, the prevalence of cognitive impairment was elevated in individuals with a BMI < 23.72 kg/m^2^, diminished significantly with increasing BMI in the 23.72–27.77 kg/m^2^ range, and exhibited an upward trend in those with a BMI > 27.77 kg/m^2^ (Figure 3). This phenomenon is demonstrated in Figure 2. Utilizing 23.72–27.77 kg/m^2^ as the reference group, the fully adjusted Model 3 shown that the prevalence of cognitive impairment in the low BMI group (<23.72 kg/m^2^) was 2.57 times that of the reference group. In contrast, the prevalence in the high BMI group (>27.77 kg/m^2^) was as high as 3.91 times that of the reference group, as illustrated in Figure 2.

### 3.4 Subgroup analyses

**Figure 4:**
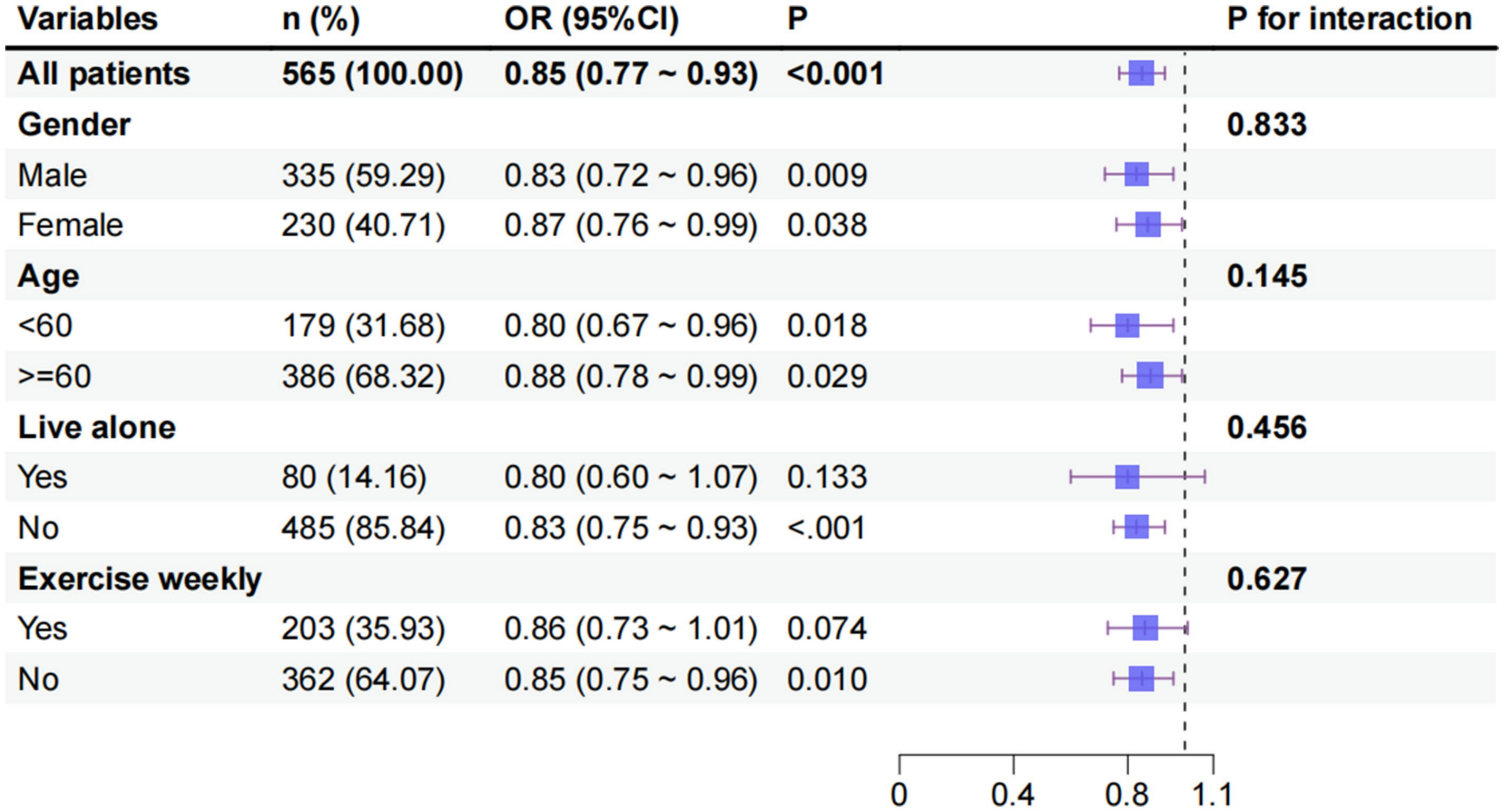
Subgroup analysis of the correlation between BMI and cognitive impairment.

Following adjustment for relevant confounders, including age, gender, residence status, marital status, exercise status, social status, smoking, drinking, PSQI, and CES-D, gender subgroup analyses demonstrated an inverse association between BMI and cognitive impairment in both the male and female subgroups (both *p* < 0.05). Subgroup analyses according to age shown that elevated BMI exhibited an inverse association with cognitive impairment in both the age <60 and age ≥60 groups (both *p* < 0.05). In the residence status subgroup, an inverse association was observed between BMI and cognitive impairment in patients with T2DM who resided alone (*p* < 0.05). Conversely, no association was found between BMI and cognitive impairment in patients with T2DM who lived with others (*p* > 0.05). Subgroup analyses of exercise demonstrated that BMI exhibited an inverse association with cognitive impairment in patients who did not exercise every week, weekly (*p* < 0.05). Conversely, no such correlation was observed between BMI and cognitive impairment in patients with T2DM who engaged in weekly exercise (*p* > 0.05). The interaction test demonstrated that the discrepancy between the stratification factors in the relationship between BMI and cognitive impairment in patients with T2DM was not statistically significant (*p*-value of the interaction test was >0.05). Refer to Figure 4 for further details.

## 4 Discussion

The comprehensive analysis of this study established a dual association between BMI and cognitive impairment. Linear analyses demonstrated an inverse association. The consistency of BMI and cognition association across adjustment levels (Model 1 OR = 0.87, 95% CI:0.81–0.95; Model 2 OR = 0.88, 95% CI:0.81–0.96; Model 3 OR = 0.84, 95% CI:0.77–0.93) confirms BMI as an independent correlate. Furthermore, RCS analyses revealed a “U”-shaped nonlinear pattern. This finding is consistent with the results of several studies conducted on middle-aged and older populations (16, 35). This pattern indicated that cognitive impairment increased with decreasing BMI at BMI values less than 23.72 kg/m^2^, reached a nadir between 23.72 kg/m^2^ and 27.77 kg/m^2^, and increased at BMI values greater than 27.77 kg/m^2^. Research conducted on middle-aged and older non-diabetic populations has demonstrated that the relationship between BMI and cognitive function is not homogeneous. Low BMI may be accompanied by muscle loss and insufficient nutrient reserves, whereas high BMI is often associated with insulin resistance and atherosclerosis, which affect neurocognitive function through different pathological pathways (16, 36–38). This ‘U-shaped’ association is more specific for middle-aged and older adults with T2DM. In T2DM, existing glucose metabolism disorders have the potential to exacerbate neurovascular damage (39). Furthermore, BMI abnormalities may further augment the risk of cognitive impairment by superimposing metabolic load (in cases of high BMI) or weakening the reserve capacity of the organism (in cases of low BMI). The cognitive advantage of the intermediate BMI range may be indicative of the relative balance between metabolic status and body reserve capacity in middle-aged and older adults with T2DM. This range may be more suitable for the physiological needs of this population.

Subgroup analysis explored the relationship between gender, age, living alone and exercise per week on cognitive impairment in T2DM patients, and the interaction between variables was not significant. The inverse association between BMI and cognitive impairment was consistent in both men and women, as well as in middle-aged and older adults with age of either <60 or ≥60 years, when stratified by sex and age. Ren et al. identified a U-shaped association between BMI and cognitive impairment in the older adult population (16). However, this association was more pronounced in men. The hypothesis proposed is that the metabolic disorders exacerbated by diabetes mellitus weakened the gender-related metabolic differences, so that the effect of BMI on cognition converged between men and women. The differential results observed between the residential status and exercise subgroups were of particular interest. The present study found an inverse association between BMI and cognitive impairment in patients residing alone, but not in those living with others. The association was found to be significant in patients who did not engage in weekly exercise, but not in those who exercised regularly. This phenomenon may be closely related to the buffering effects of social support and behavioral interventions. The present study hypothesizes that middle-aged and older adults with T2DM who reside with others are more likely to receive life support, including meal management and medication reminders. It is also postulated that increased social interaction may offer a degree of indirect protection for neurological function by improving mood states and maintaining cognitive stimulation, thereby weakening the effect of BMI on cognitive functioning (40). Conversely, regular exercise has been demonstrated to have a positive impact on cognitive function, with studies indicating that it may enhance insulin sensitivity, promote cerebral perfusion, and upregulate the expression of neurotrophic factors. These protective effects of exercise may potentially counterbalance the risks associated with BMI abnormalities (41). This finding is consistent with the results of numerous studies, which have demonstrated that social participation and regular exercise are independent protective factors for cognitive function in middle-aged and older adults (42–44). Furthermore, it has been shown that the effects of these activities may partially offset the adverse effects of body mass-related indicators. This finding indicates that the regulation of body mass may represent a viable strategy for preserving cognitive function in middle-aged and older adults with T2DM residing alone and exhibiting physical inactivity.

Despite the subgroup analyses demonstrating some disparities, the interaction test revealed that the stratification factors did not exert a statistically significant modifying effect on the observed association pattern. This suggests that the observed subgroup differences in this study may have been influenced by limitations in sample size or the absence of measurement of other confounders (e.g., dietary structure, severity of the underlying disease). These limitations require validation in a larger sample study. Moreover, these findings imply that the fundamental association between BMI and cognitive function remains relatively consistent in middle-aged and older adults patients with T2DM. This finding indicates that BMI should be considered a significant influence factor when assessing cognitive function in T2DM patients. These findings provide a framework for future intervention studies to enhance cognitive function in T2DM patients by adjusting BMI (e.g., BMI monitoring in inactive patients or those living alone).

Despite the incorporation of inclusion and exclusion criteria, as well as statistical modeling, to mitigate the influence of potential confounders, it is acknowledged that the outcomes of the study may be susceptible to unmeasured or uncontrolled confounders (e.g., specific complications, medications, and blood glucose control). Subsequent studies must incorporate more rigorous control for these factors to ensure the validity of the results. This study was of a cross-sectional nature. It is therefore recommended that prospective cohort studies be conducted in the future in order to elucidate further the potential causal relationship between BMI and cognitive impairment in T2DM. In addition, Future studies may apply sensitivity analyses with alternative variable categorizations to further validate findings. Crucially, the residential status and exercise effects emerged as exploratory rather than predetermined findings. Their mechanistic plausibility warrants validation in dedicated hypothesis-driven studies.

## 5 Conclusion

The present study revealed a nonlinear relationship and subgroup differences between BMI and cognitive dysfunction in middle-aged and older adults with T2DM. These findings highlight the importance of maintaining an optimal BMI range and the potential benefits of social support and physical activity in managing cognitive dysfunction. Therefore, it is recommended that T2DM patients maintain a BMI within the optimal range (23.72–27.77 kg/m^2^) and engage in regular physical activity and social support programs. These recommendations could be integrated into clinical practice, community health initiatives, and public health policies to improve the management of cognitive dysfunction in T2DM patients. However, it is important to acknowledge that our cross-sectional design limits our ability to establish causality, and the results may be affected by unmeasured variables. In conclusion, addressing the complex interplay between BMI, cognitive dysfunction, and lifestyle factors in T2DM patients could have significant implications for public health, research, and professional training, potentially leading to improved quality of life and reduced cognitive decline in this vulnerable population.

## Data Availability

The raw data supporting the conclusions of this article will be made available by the authors, without undue reservation.
